# Research on Aerial Autonomous Docking and Landing Technology of Dual Multi-Rotor UAV

**DOI:** 10.3390/s22239066

**Published:** 2022-11-22

**Authors:** Liang Wang, Xiangqian Jiang, Di Wang, Lisheng Wang, Zhijun Tu, Jianliang Ai

**Affiliations:** 1 Department of Aeronautics and Astronautics, Fudan University, Shanghai 200433, China; 2 Shanghai Aerospace Systems Engineering Institute, Shanghai 201108, China

**Keywords:** multi-UAV, autonomous transportation, RBF−PID, flight control, machine vision, image processing, target identification, trajectory tracking, information fusion

## Abstract

This paper studies the cooperative control of multiple unmanned aerial vehicles (UAVs) with sensors and autonomous flight capabilities. In this paper, an architecture is proposed that takes a small quadrotor as a mission UAV and a large six-rotor as a platform UAV to provide an aerial take-off and landing platform and transport carrier for the mission UAV. The design of a tracking controller for an autonomous docking and landing trajectory system is the focus of this research. To examine the system’s overall design, a dual-machine trajectory-tracking control simulation platform is created via MATLAB/Simulink. Then, an autonomous docking and landing trajectory-tracking controller based on radial basis function proportional–integral–derivative control is designed, which fulfills the trajectory-tracking control requirements of the autonomous docking and landing process by efficiently suppressing the external airflow disturbance according to the simulation results. A YOLOv3-based vision pilot system is designed to calibrate the rate of the aerial docking and landing position to eight frames per second. The feasibility of the multi-rotor aerial autonomous docking and landing technology is verified using prototype flight tests during the day and at night. It lays a technical foundation for UAV transportation, autonomous take-off, landing in the air, and collaborative networking. In addition, compared with the existing technologies, our research completes the closed loop of the technical process through modeling, algorithm design and testing, virtual simulation verification, prototype manufacturing, and flight test, which have better realizability.

## 1. Introduction

**Figure 1:**
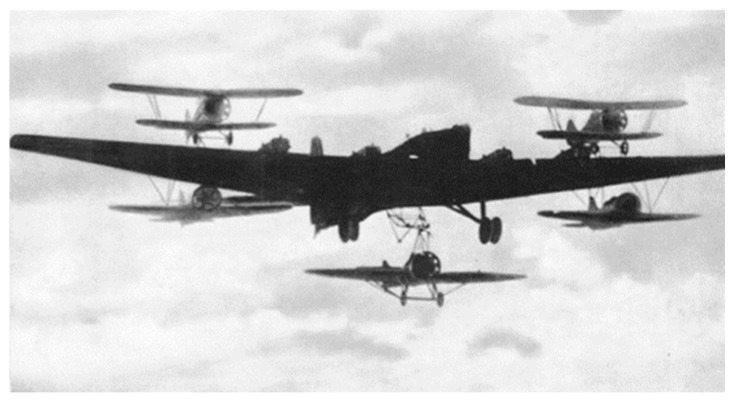
The Soviet Zveno-1 “mother plane” made its first flight in 1931.

The helicarrier has appeared in science fiction films in recent years. The one in S.H.I.E.L.D., for example, is a helicarrier in the form of a massive platform for sea, ground, and air, powered by four large turbine engines [1]. The mothership carries a small aircraft that can take off and land at the same time. The idea of an “aircraft carrier” is not new, as the notion of releasing small planes from large ones has existed for a long time. Since the advent of aircraft carriers, many researchers have explored “aircraft carriers” that can fly in the sky. From the 1930s to the 1950s, the United States, Soviet Union, Germany, and other aviation powers developed a “mother aircraft” one after another. The Soviet Zveno-1 “mother plane”, which made its first flight in 1931, was a TB-1 aircraft carrying two aircraft [2], as shown in Figure 1. In June 1932, Curtis converted the airships AKRON and MACON into airships capable of carrying up to three aircraft [3].

At present, an “aircraft carrier” that can recover and release manned aircraft, as seen in science fiction movies, is difficult to achieve, but the maturity of drone technology has opened another avenue of research. With the rapid development of drone technology at present, many researchers are actively developing “drone carriers” that can take drones out of the air and return them.

On 15 December 2014, DARPA issued a request for information-seeking technology to use large aircraft as carriers to transport, launch, and recover small UAVs, each weighing up to 45 kg. Prior to the successful recovery, DARPA launched a series of test flights starting in October 2020, with nine attempts to retrieve three aircraft, all of which failed, but through which they gained a wealth of experience [4]. Subsequently, UAVs are the main force of the “air mother ship”, and with the help of platform UAVs’ longer aloft time and cruising range, the mission UAVs’ aloft time and cruising range will be greatly extended. They can not only conduct missions far away from the base but also deploy forward with the help of the “air mother ship” and greatly shorten the reaction time of UAVs. The combination of large-platform UAVs and small-mission UAVs can further enhance the multi-purpose capability of UAVs.

In this article, a cluster technical scheme is adopted to design a platform UAV that can carry a mission UAV. A small quadrotor is used as the mission UAV and a large hexacopter UAV is used as the mission UAV take-off and landing platform; the hexacopter is also the transport carrier of the mission UAV. Autonomous docking and landing technology in the air was the technical difficulty of this project. Through a literature survey, we found some relevant research papers. Tao Liu et al. proposed a model-based distributed algorithm to minimize the energy consumption of the multi-UAV system, in which each UAV obtains its trajectory by solving the corresponding sub-problem, and the UAVs coordinate their trajectories according to the master problem [5]. Due to the application-specific nature of wireless sensor networks, Bhisham Sharma et al. proposed a reliable and congestion-based protocol, which provides both bidirectional reliability and rate-adjustment-based congestion control [6]. Thien-Minh Nguyen et al. proposed a method combining an ultra-wideband (UWB) ranging sensor with vision-based techniques to achieve both autonomous approaching and landing capabilities in GPS-denied environments [7]. Chengbin Chen et al. proposed a deep learning-based energy optimization algorithm (DEO) to dynamically adjust the emission energy of the ED so that the received energy of the mobile relay UAV is, as much as possible, equal to the sensitivity of the receiver [8]. In this paper, the research of autonomous docking and landing trajectory-tracking control algorithms for mission UAVs and platform UAVs is presented. The trajectory tracking control simulation platform of dual multi-rotor UAVs is constructed by MATLAB/Simulink, and the parameters of the dynamic system are solved, with the expected aircraft trajectory being used to verify the control algorithm. The principle, design method, and implementation process of the RBF-PID control trajectory tracking controller are described, and various response curves of the controller under the control of mission UAV trajectory-tracking control and airflow disturbance are obtained by simulation. The design process of the visual guidance system based on YOLOv3 is described, and the architecture of the aerial autonomous docking and landing control system is constructed. Through a large number of flight tests to continuously optimize the program, the calibration rate of the aerial docking and landing position reached 8FPS, which met the requirements of autonomous aerial docking and landing and performed well in both daytime and nighttime flight tests.

## 2. Modeling of Mission UAV and Platform UAV

**Figure 2:**
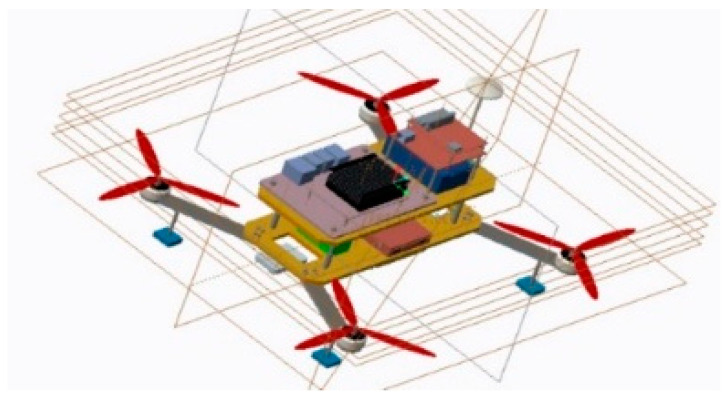
Three-dimensional model of mission UAV.

**Figure 3:**
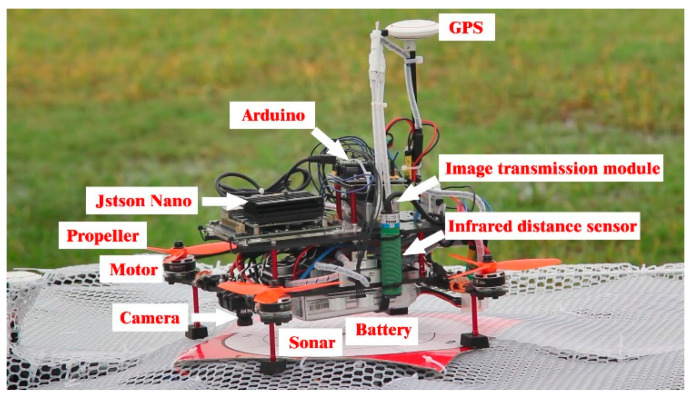
Real mission UAV and its components.

**Figure 4:**
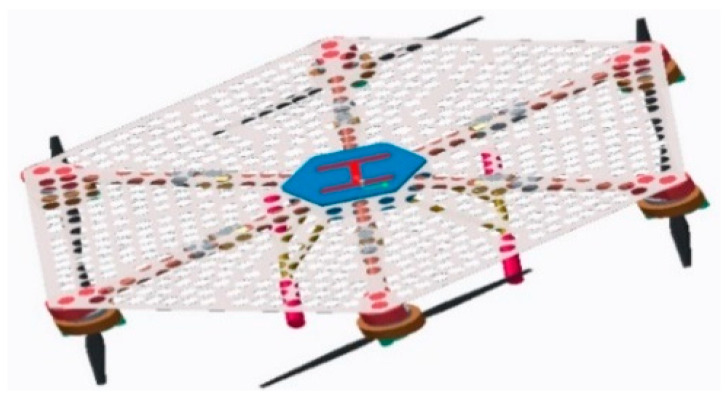
Three-dimensional model of the platform UAV.

**Figure 5:**
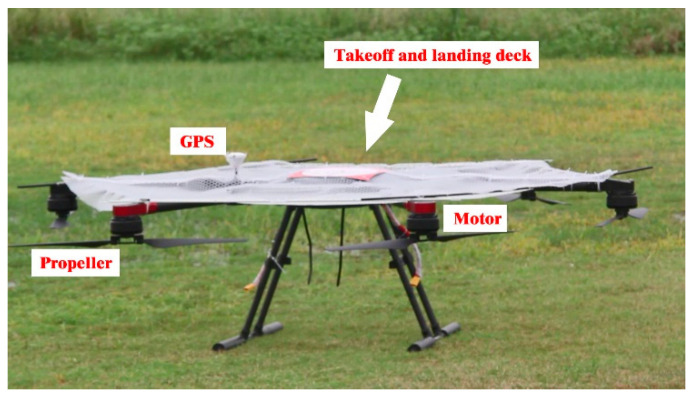
Real platform UAV.

**Figure 6:**
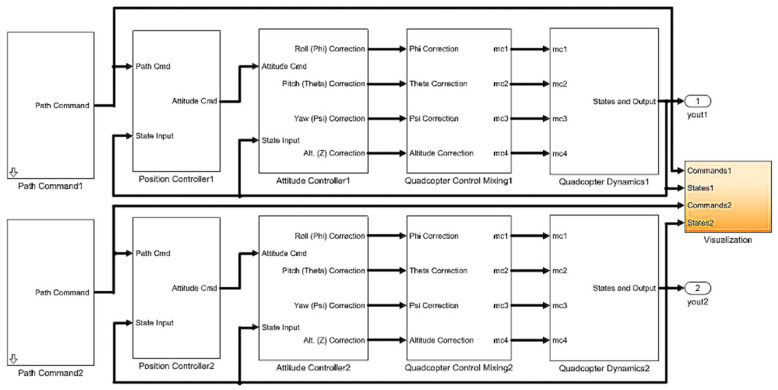
Flight simulation environment.

By analyzing the mission requirements in detail, the mission UAV and the platform UAV were designed. First, the mathematical models were derived and established by analyzing the frame design, airborne equipment type selection, and power system matching [9,10]. Based on this analysis, an experimental prototype was fabricated to verify the aerial autonomous docking, take-off, and landing technology, as shown in Figure 2, Figure 3, Figure 4 and Figure 5. Figure 6 shows the designed flight simulation environment.

The flight simulation environment based on MATLAB Simulink aims to verify the dynamic performance of the aircraft and test the dual multi-rotor UAV trajectory tracking and dynamic docking performance in the simulation environment, that is, the mission UAV docking and landing on the platform UAV in flight. The simulation platform is divided into three parts: The mission UAV simulation module, the platform UAV simulation module, and the visualization module, as shown in Figure 6. The simulation part of the mission UAV includes a path-generation module, a position controller module, an attitude controller module, a quadrotor hybrid control module, a quadrotor dynamics module, feedback, and output. The simulation part of the platform UAV includes a path-generation module, a position controller module, an attitude controller module, a six-rotor hybrid control module, a six-rotor dynamics module, feedback, and output.

## 3. Mathematical Modeling of the Mission UAV

### 3.1. Definition of the Coordinate Systems

A suitable coordinate system should be established to calculate and express the attitude and position of the UAV (unmanned aerial vehicle) more accurately. It also contributes to clarifying the relationship between the variables and deducing the state equation of precisely describing the flight characteristics of the designed rotorcraft UAV [11]. Then, design research is unfolded step by step by establishing a complete simulation platform for the autonomous docking and landing of the two aircraft.

#### 3.1.1. Geographical Coordinate System $O_{g}x_{g}y_{g}z_{g}$

The origin $O_{g}x_{g}y_{g}z_{g}$ of the geographic coordinate system (denoted as $S_{g}$, the inertial coordinate system) coincides with the centroid of the multi-rotor UAV (the centroid of the multi-rotor UAV coincides with its geometric center upon the optimized layout of airborne equipment); the positive direction of the $O_{g}x_{g}$ and $O_{g}y_{g}$ axes in the horizontal plane point to the north and the west, respectively; and the $O_{g}z_{g}$ axis is perpendicular to the horizontal plane.

#### 3.1.2. Airframe Coordinate System $O_{b}x_{b}y_{b}z_{b}$

**Figure 7:**
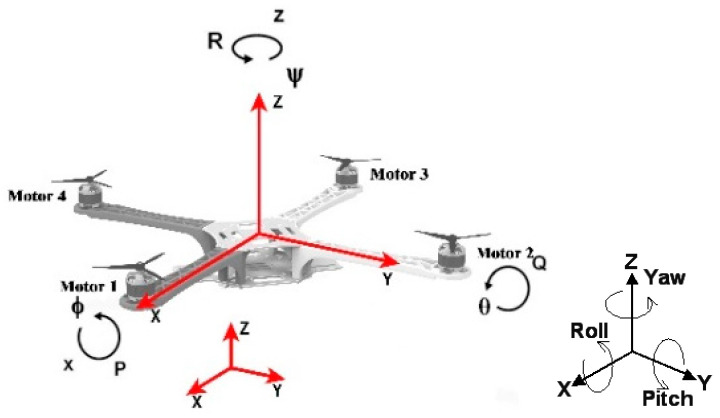
Coordinate axis direction setting (arrow direction is positive (right rotation)).

The origin $O_{b}$ of the airframe coordinate system $O_{b}x_{b}y_{b}z_{b}$ (denoted as $S_{b}$) coincides with the centroid of the multi-rotor UAV. Specifically, the $O_{b}x_{b}$ axis coincides with the longitudinal axis of the multi-rotor UAV with the positive direction pointing forward; the $O_{b}z_{b}$ axis is perpendicular to the horizontal plane of the multi-rotor UAV airframe with the positive direction pointing right above; and $O_{b}x_{b}y_{b}z_{b}$ is perpendicular to the $O_{b}x_{b}z_{b}$ plane with its positive direction determined by the right-hand rule. As shown in Figure 7.

The following settings were applied before the establishment of the UAV’s mathematical model: 

Setting 1: The Earth’s surface was assumed to be planar, with the influence of Earth’s rotation and curvature ignored.

Setting 2: An UAV is regarded as a rigid body since elastic deformation in the flight course does not occur during flight.

Setting 3: The load of the UAV does not change during flight.

Setting 4: The overall centroid coincides with the origin of the airframe coordinate system, with the structure and mass distribution being symmetrical after the layout of airborne equipment is optimized with the particle swarm algorithm.

The x-axis was set as the forward direction of the UAV motion. The direction of the coordinate axis was set as below: 

### 3.2. Matrix of Mass Moment of Inertia

The mass moment of inertia of the four-axis aircraft on the defined axis was described using the inertia matrix, which is essential for the systemic analysis of flight dynamics. With some approximations, the mass moment of inertia on the x-, y-, and z-axes can be determined to improve the required inertia matrix. 

With an “X” structure adopted as the rack, the inertia matrix can be expressed by Equation (1):

(1) $$J^{b}=\left[\begin{array}{ccc} J_{xx} & 0 & 0\\ 0 & J_{yy} & 0\\ 0 & 0 & J_{zz} \end{array}\right]$$

$J^{b}$ is the inertia of the quadcopter relative to the airframe, where $J_{xx}$, $J_{yy}$, and $J_{zz}$ represent the inertia of the quadcopter on each axis, respectively.

### 3.3. Inertia Moment Calculation

The rotational inertia of the mission UAV was calculated in four parts, including the rotational inertia of the motor, the rotational inertia of the electronic speed controller, the rotational inertia of the UAV central equipment board (containing airborne equipment), and the rotational inertia of the manipulator.

a. Calculation of the inertia moment of the motor:

(2) $$J_{x,M}=J_{y,M}=2\left[\frac{1}{4}m_{\mathrm{motor}}r^{2}+\frac{1}{3}m_{\mathrm{motor}}h^{2}\right]+2\left[\frac{1}{4}m_{\mathrm{motor}}r^{2}+\frac{1}{3}m_{\mathrm{motor}}h^{2}+m_{\mathrm{motor}}d_{\mathrm{motor}}^{2}\right]$$

(3) $$J_{z,M}=4\left[\frac{1}{2}m_{\mathrm{motor}}r^{2}+m_{\mathrm{motor}}d_{\mathrm{motor}}^{2}\right]$$

where $m_{\mathrm{motor}}$ is the mass of the motor; $d_{\mathrm{motor}}^{}$ is the distance from the motor to the origin of the airframe coordinate system; E is the motor height; and r is the motor radius.

b. Calculation of the inertia moment of the electronic speed controller (ESC):

(4) $$J_{x,S}=J_{y,S}=2\left[\frac{1}{12}m_{\mathrm{ESC}}a^{2}\right]+2\left[\frac{1}{12}m_{\mathrm{ESC}}b^{2}+m_{\mathrm{ESC}}d_{\mathrm{ESC}}^{2}\right]$$

(5) $$J_{z,S}=4\left[\frac{1}{12}m_{\mathrm{ESC}}\left(a^{2}+b^{2}\right)+m_{\mathrm{ESC}}d_{\mathrm{ESC}}^{2}\right]$$

where $m_{\mathrm{ESC}}$ is the mass of the electronic speed controller; $d_{\mathrm{ESC}}^{}$ is the distance from the electronic speed controller to the origin of the airframe coordinate system; a is the width of the electronic speed controller; and b is the length of the electronic speed controller.

c. Calculation of the inertia moment of the central equipment module (CEM) (including airborne equipment):

(6) $$J_{x,H}=J_{y,H}=\left[\frac{1}{4}m_{\mathrm{CEM}}r_{\mathrm{CEM}}{}^{2}+\frac{1}{12}m_{\mathrm{CEM}}H^{2}\right]$$

(7) $$J_{Z,H}=\left[\frac{1}{2}m_{\mathrm{CEM}}r_{\mathrm{CEM}}{}^{2}\right]$$

where $m_{\mathrm{CEM}}$ is the mass of the central equipment module; E is the radius of the equivalent cylinder of the central equipment module; and H is the height of the equivalent cylinder of the central equipment module.

d. Calculation of the inertia moment of the arm:

(8) $$J_{x,A}=J_{y,A}=2\left[\frac{1}{2}m_{\mathrm{arm}}r_{\mathrm{arm}}{}^{2}\right]+2\left[\frac{1}{4}m_{\mathrm{arm}}r_{\mathrm{arm}}{}^{2}+\frac{1}{3}m_{\mathrm{arm}}L^{2}+m_{\mathrm{arm}}d_{\mathrm{arm}}^{2}\right]$$

(9) $$J_{Z,H}=4\left[\frac{1}{2}mr_{\mathrm{arm}}{}^{2}\right]$$

where $m_{\mathrm{arm}}$ is the mass of the quadcopter arm; $r_{\mathrm{arm}}$ is the radius of the quadcopter arm; *L* is the length of the quadcopter arm; and $d_{\mathrm{arm}}$ is the distance from the end of the quadcopter arm to the z-axis.

### 3.4. Thrust Coefficient

The thrust T provided by a single-motor system can be calculated as

(10) $$T=C_{T}\rho A_{r}r^{2}\varpi^{2}$$

*C_r_* is the thrust coefficient of a given motor; *ρ* is the air density; *A_r_* is the cross-sectional area of the propeller rotation; *r* is the rotor radius; and *ϖ* is the angular velocity of the rotor. Additionally, the thrust provided by the motor provides forces perpendicular to the X–Y plane of the airframe in the positive z direction. 

### 3.5. Torque Coefficient


(11) $$Q=c_{Q}\varpi^{2}$$

Q is the torque generated by the motor and $c_{Q}$ is the torque coefficient of the motor system. It provides a force that deflects the system around the z-axis.

### 3.6. Initial Matrix Structure

A matrix was created to describe the thrust and moment on the system. $d_{+}$ is the distance between the motor and its axis of rotation. $d_{+}$ is the length from the motor center to the manipulator length of the motor in quadcopters. Additionally, $d_{x}$ can be replaced by $d_{x}\sin(45^{\circ })$ if X is adopted for the configuration. Hence, the configuration adjustment exerts no impact on $c_{Q}$, while the effect of $c_{T}$ is distributed over the pitch and roll amounts of all 4 motors.

(12) $$\left[\begin{array}{c} \Sigma T\\ \tau_{\phi }\\ \tau_{\theta }\\ \tau_{\psi } \end{array}\right]=\left[\begin{array}{cccc} c_{T} & c_{T} & c_{T} & c_{T}\\ -d_{x}c_{T} & d_{x}c_{T} & d_{x}c_{T} & -d_{x}c_{T}\\ -d_{x}c_{T} & -d_{x}c_{T} & d_{x}c_{T} & d_{x}c_{T}\\ -c_{Q} & c_{Q} & -c_{Q} & c_{Q} \end{array}\right]\left[\begin{array}{c} \varpi_{1}^{2}\\ \varpi_{2}^{2}\\ \varpi_{3}^{2}\\ \varpi_{4}^{2} \end{array}\right]$$

### 3.7. Relationship between the Motor Control Signal and Output Speed Command

The coefficients of thrust and moment are crucial to achieving the control purpose. It is the relationship with the motor speed that exerts control over, rather than directly being determined by, the control system (such as the throttle command). In that case, the control signal (PWM) command value should be converted into a rotational speed (RPM) value using linear regression. The following equation is created.

(13) $$\varpi_{ss}=(\mathrm{Throttle}\%)c_{R}+\varpi_{b}$$

$\varpi_{ss}$ is the expected steady motor speed and throttle valve percentage command; *c_R_* is the conversion coefficient of throttle percentage and RPM; and $\varpi_{b}$ is the y-axis intercept of the linear regression relationship.

### 3.8. Gyroscopic Moment

The gyroscope force generated on the airframe is controlled by the inertia $J_{m}$, rolling rate P, and pitch rate Q of the rotating parts of each motor, as well as the speed $w_{i}$ of each motor system. The pitch and roll moments generated by the motors are expressed by Equation (14):

(14) $$\begin{array}{l} \tau_{\phi_{gyro}}=J_{m}Q\left(\frac{\pi }{30}\right)\left(w_{1}-\varpi_{2}+\varpi_{3}-\varpi_{4}\right)\\ \tau_{\theta_{gyro}}=J_{m}P\left(\frac{\pi }{30}\right)\left(-\varpi_{1}+\varpi_{2}-\varpi_{3}+\varpi_{4}\right) \end{array}$$

### 3.9. Final Matrix Structure

By adding all the motor-propeller forces to the equations, the equations can be organized into a matrix form for simulation. The relational expressions of the aerodynamic moment, gyroscope moment, and thrust moment are

(15) $$M_{A,T}^{b}=\left[\begin{array}{c} d_{+}c_{T}\varpi_{2}^{2}-d_{+}c_{T}\varpi_{4}^{2}+J_{m}Q\left(\frac{\pi }{30}\right)\left(\varpi_{1}-\varpi_{2}+\varpi_{3}-\varpi_{4}\right)\\ -d_{+}c_{T}\varpi_{1}^{2}+d_{+}c_{T}\varpi_{3}^{2}+J_{m}P\left(\frac{\pi }{30}\right)\left(-\varpi_{1}+w_{2}-\varpi_{3}+w_{4}\right)\\ -c_{Q}\varpi_{1}^{2}+c_{Q}\varpi_{2}^{2}-c_{Q}\varpi_{3}^{2}+c_{Q}\varpi_{4}^{2} \end{array}\right]$$

where $M_{A,T}^{b}$ is the framed moment due to the aerodynamics, thrust, and system moments.

The body of the quadcopter is also affected by gravity and rotor lift. The lift force can be expressed as

(16) $$F_{A,T}^{b}=\left[\begin{array}{c} 0\\ 0\\ c_{T}\left(\varpi_{1}^{2}+\varpi_{2}^{2}+\varpi_{3}^{2}+\varpi_{4}^{2}\right) \end{array}\right]$$

$F_{A,T}^{b}$ refers to the force of aerodynamic forces and thrust (assumed to be strictly positive in the z-direction) acting on the airframe of the quadcopter.

### 3.10. Equation of State

The equation of the state of angular velocity is

(17) $${}^{b}{\dot{\omega }}_{b|i}^{b}={\left(J^{b}\right)}^{-1}\left[M_{A,T}^{b}-\Omega_{b|j}^{b}J^{b}\omega_{b|i}^{b}\right]=\left[\begin{array}{c} \dot{P}\\ \dot{Q}\\ \dot{R} \end{array}\right]$$

Changes in the roll (P), pitch (Q), and yaw (R) rates of the quadcopter are described in Equation (17) with considerations of the inertia, angular velocity, and moment applied by the motor-propeller system. ${}^{b}{\dot{\omega }}_{b|i}^{b}$ is the angular acceleration of each axis in the airframe coordinate system relative to the inertial coordinate system. P, Q and R are the speeds of rotation around the X, Y, and Z axes, respectively.

The Euler equation of kinematics (18) is the equation of state to determine the rate of change of Euler angles in the inertial frame.

(18) $$\dot{\Phi }=H(\Phi )\omega_{b|i}^{b}=\left[\begin{array}{c} \dot{\phi }\\ \dot{\theta }\\ \dot{\psi } \end{array}\right]$$

The angular velocity of the aircraft in the airframe can be associated with the change in angular rotation using the aerospace sequential rotation matrix, as shown in Equation (19):

(19) $$\omega_{b|i}^{b}=\left[\begin{array}{c} \dot{\phi }\\ 0\\ 0 \end{array}\right]+C_{\phi }\left(\left[\begin{array}{l} 0\\ \dot{\theta }\\ 0 \end{array}\right]+C_{\theta }\left[\begin{array}{l} 0\\ 0\\ \dot{\psi } \end{array}\right]\right)$$

The Euler equation of kinematics can be detected by derivation upon performing matrix multiplication and addition:

(20) $$\dot{\Phi }=\left[\begin{array}{l} \dot{\phi }\\ \dot{\theta }\\ \dot{\psi } \end{array}\right]=\left[\begin{array}{ccc} 1 & t(\theta )s(\phi ) & t(\theta )c(\phi )\\ 0 & c(\phi ) & -s(\phi )\\ 0 & s(\phi )/c(\theta ) & c(\phi )/c(\theta ) \end{array}\right]\left[\begin{array}{c} P\\ Q\\ R \end{array}\right]=H(\Phi )\omega_{b|i}^{b}$$

The equation of the velocity state is shown in Equation (21):

(21) $$b_{\dot{V}}{}_{CM|i}^{b}=\left(\frac{1}{m}\right)F_{A,T}^{b}+g^{b}-\Omega_{b|i}^{b}\omega_{CM|i}^{b}=\left[\begin{array}{c} \dot{U}\\ \dot{V}\\ \dot{W} \end{array}\right]$$

$b_{\dot{V}}{}_{CM|i}^{b}$ is the linear acceleration of the center of mass relative to the inertial coordinate system in the airframe coordinate system. The variable *m* is the total mass of the quadcopter and $g^{b}$ is the gravitational acceleration of the rotation matrix $C_{b|i}$ acting on the airframe.

(22) $$g^{b}=C_{b|i}g^{i}$$

The equation of position state is

(23) $${}^{i}{\dot{P}}_{CM|i}^{i}=C_{i|b}v_{CM|i}^{b}=\left[\begin{array}{c} \dot{X}\\ \dot{Y}\\ \dot{Z} \end{array}\right]$$

where $v_{CM|i}^{b}$ is rotated to the inertial coordinate system via the transposed $C_{b|i}$ by the speed of the quadcopter in the airframe coordinate system, which is $C_{b|i}$.

## 4. Architectural Design of Aerial Autonomous Docking and Landing Control System 

**Figure 8:**
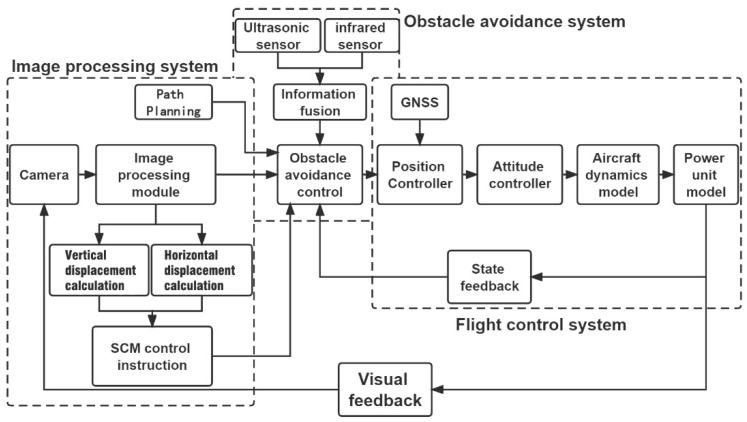
Architectural design of the aerial autonomous docking and landing control system.

Figure 8 shows the architectural design of the aerial autonomous docking and landing control system. First, the video information stream is sent to the image processing module by image acquisition to identify, locate, and track the landing area of the platform UAV. The data obtained from positioning after calculating the vertical and horizontal displacements are sent to the single-chip microcomputer module to generate the expected trajectory control signal of the vertical and horizontal displacements of the mission UAV. Next, the control signal obtained is input into the obstacle avoidance system to safeguard the UAV flight environment; the signal is also input into the underlying flight control system to control the mission UAV for the expected trajectory-tracking movement. The visual feedback is conducive to quickly updating the location of the platform UAV and updating the rate in real-time, which is the loop execution rate for the autonomous docking and landing control system.

## 5. Eight-Frames-per-Second Trajectory-Tracking Controller

The network of the radial basis function (RBF), as a high-performance artificial neural network, was established for abstracting the mechanism of biological local regulation, which can satisfy the requirements of detection and recognition in specific fields well. Moreover, RBF, seen as a two-layer forward network, can simulate the local adjustment of the nervous system [12]. Additionally, it has obvious advantages in the local approximation, such as the accurate approximation of nonlinear functions according to requirements.

**Figure 9:**
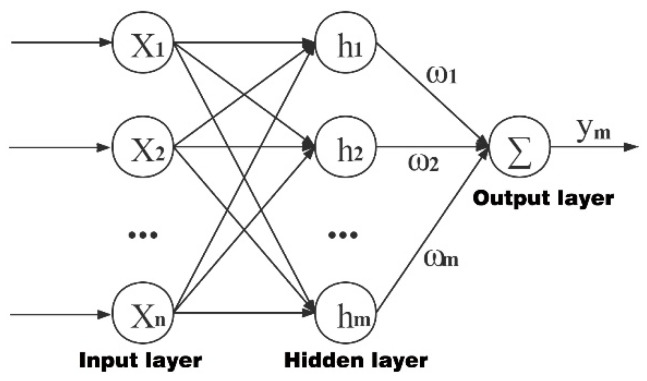
RBF network structure.

The signal source node can identify the appropriate number of hidden layer elements and related node parameters as the input of this network, according to the target problem [13]. The composition of a three-layer RBF network is shown in the following Figure 9. Evidently, *N* input nodes, *P* implicit nodes, and an input node are included in this system, entered as $X={\left[x_{1},x_{2},\ldots ,x_{N}\right]}^{T}$.

As can be seen from Figure 9, the RBF network is composed of the following:

Hidden layer: For nonlinear mapping,

(24) $$X\to h_{j}(x)=f_{j}\left(\frac{\|X-C_{j}\|}{b_{j}}\right)$$

Basis function:

(25) $$h_{j}(x)=f_{j}\left(\frac{\left\|X-C_{j}\right\|}{b_{j}}\right),\;j=1,2,\cdots ,P$$

where $C_{j}={\left[c_{j1},c_{j2},\cdots ,c_{ji},\cdots ,c_{jN}\right]}^{T}(i=1,2,\cdots ,N)$ is a specific j node of the center vector; $b_{j}$ is the width vector of the j node, which is a positive number and can be selected as per the width requirements; $B={\left[b_{1},b_{2},\cdots ,b_{j},\cdots ,\cdots ,b_{m}\right]}^{T}$ is the width vector of the entire network; $\left\|X-C_{j}\right\|$ is the norm of $X-C_{j}$, which is used to measure the distance between the two; *f_j_* can be regarded as a function of radial symmetry, with its value being negatively correlated with $\left\|X-C_{j}\right\|$. For the given input X, a few units near the center are activated [12]. 

Output layer: For the nonlinear mapping of $h_{j}(x)\to y_{m}$,

(26) $$y_{m}(k)=w_{1}h_{1}+w_{2}h_{2}+\cdots +w_{m}h_{m}$$

where $w={\left[w_{1},w_{2},\cdots ,w_{m}\right]}^{T}$ corresponds to the network output weight vector in the above equation.

The Gaussian function is the most used:

(27) $$h_{j}(x)=f_{j}\left(\frac{\left\|X-C_{j}\right\|}{b_{j}}\right)=\exp\left(-\frac{{\left\|X-C_{j}\right\|}^{2}}{b_{j}^{2}}\right),j=1,2\ldots m$$

A detailed analysis of the above equation shows that the output value of the node is limited to (0, 1). The small distance between the input value and the center results in a large output with the C value closely related to its influence under the circumstance of this network processing. Moreover, the network smoothness is improved with the increase in its value, while the shape of the function is narrow in the opposite case. Hence, the comparative analysis shows that the output tends to be 1 only if the input is quite close to the weight vector. Furthermore, the advantages of the Gaussian basis function are shown in several aspects, such as the simple form, convenient analysis and processing, the moderate increase in the system difficulty under the condition of multivariate input, and good smoothness and certain symmetry [14]. On this basis, the corresponding application difficulty is significantly reduced, and each derivative can be easily determined in processing. In addition, this function is also convenient for theoretical analysis. However, it is also defective in some respects, such as a lack of compactness. As a result, the weights cannot be adjusted locally in the application. The $h_{j}(x)$ value is at a very low level and can be approximated to 0 when it is far away, according to the application results. Conversely, the weight $w_{ij}$ should be modified when $h_{j}(x)$ reaches certain conditions. Hence, it is expected that the network performance can be improved to a certain extent after optimization, with better approximation and improved learning function [15].

Additionally, the following nonlinear radial functions can also be selected:
Thin plate spline$f(x)=x^{2}\log_{2}x$(1)Cubic function$f(x)=x^{3}$(2)Multiple quadratic function$f(x)={\left(x^{2}+c^{2}\right)}^{k},0<k<1$(3)Inverse multiple quadratic function$f(x)={\left(x^{2}+c^{2}\right)}^{-k},0<k<1$(4)

The characteristics of the target problem in the application process were necessarily analyzed and a specific radial basis function was selected because of obvious differences existing in the performance of the RBF network. The Gaussian function was selected for comparative analysis, so as to better satisfy the application requirements and improve the learning performance of the network. Furthermore, it can also improve the applicability of the system. Additionally, any continuous nonlinear function can be approximated in the application process. Additionally, it differs from simple neural networks in that the functions are used differently. The weighting coefficients of the RBF network should be adjusted during processing, causing the processing speed to be significantly reduced [16]. This is also true in real-time. Therefore, its further application and promotion should be supported with the appropriate improvement. The comparative analysis shows that there is no such defect in the RBF network. Small weight values are required for various input and output data pairs. In that case, the learning and training speed can be enhanced markedly.

The center value, width, and output weight of the Gaussian function are parameters that should be adjusted in the application of the RBF network.

The output of the radial basis vector $H={\left[h_{1},h_{2},\ldots h_{P}\right]}^{T}$ of the identification network was set.

The performance indicator function of the RBF neural network is

(28) $$J_{O}(k)=\frac{1}{2}{\left[y(k)-y_{m}(k)\right]}^{2}$$

Since identification results are determined directly by the target Jacobian function, an inertia term should be introduced for revision to better meet the requirements of the RBF identification performance. Additionally, the width and output weight coefficients were modified using the gradient descent method. The corresponding expressions were modified as

(29) $$\begin{array}{c} \Delta w_{j}=\left[y(k)-y_{m}(k)\right]h_{j}\\ w_{j}(k)=w_{j}(k-1)+\eta_{0}\Delta w_{j}+\alpha_{0}\left[w_{j}(k-1)-w_{j}(k-2)\right]+\\ \beta_{0}\left[w_{j}(k-2)-w_{j}(k-3)\right] \end{array}\}$$

(30) $$\begin{array}{c} \Delta b_{j}=\left[y(k)-y_{m}(k)\right]w_{j}h_{j}\frac{{\left\|X-C_{j}\right\|}^{2}}{b_{j}^{3}}\\ b_{j}(k)=b_{j}(k-1)+\eta_{0}\Delta b_{j}+\alpha_{0}\left[b_{j}(k-1)-b_{j}(k-2)\right]+\\ \beta_{0}\left[b_{j}(k-2)-b_{j}(k-3)\right] \end{array}\}$$

(31) $$\begin{array}{c} \Delta c_{ji}=\left[y(k)-y_{m}(k)\right]w_{j}\frac{x_{j}-c_{ji}}{b_{j}^{2}}\\ c_{ji}(k)=c_{ji}(k-1)+\eta_{0}\Delta c_{ji}+\alpha_{0}\left[c_{ji}(k-1)-c_{ji}(k-2)\right]+\\ \beta_{0}\left[c_{ji}(k-2)-c_{ji}(k-3)\right] \end{array}\}$$

where $\eta_{0}$ is the learning rate and $\alpha_{0}$ and $\beta_{0}$ are inertia coefficients, $\eta_{0},\alpha_{0},\beta_{0}\in (0,1)$. The Jacobian formula of the output result to the sensitivity of the input is

(32) $$\frac{\partial y(k)}{\partial \Delta u(k)}\approx \frac{\partial y_{1}(k)}{\partial \Delta u(k)}=\sum_{j=1}^{P}w_{j}h_{j}\frac{c_{ji}-x_{1}}{b_{j}^{2}}$$

**Figure 10:**
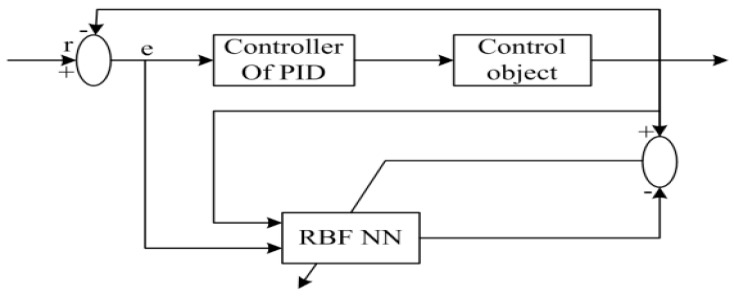
Adaptive PID control system based on the RBF neural network.

As shown in Figure 10, the neural network controller was composed of two parts:

1. Neural network controller. Incremental PID controller, four-rotor trajectory-tracking closed-loop control, and online PID tuning of $k_{p}$, $k_{i}$, and $k_{d}$;

2. PID controller parameters are adjusted by the RBF neural network controller through neural network self-learning to achieve an optimal control performance.

The control error of the incremental PID controller is

(33) error(k)=r(k)−y(k)

The proportional term, integral term, and differential term in the PID control algorithm can be expressed as

(34) $$\begin{array}{l} xc(1)=\mathrm{error}(k)-\mathrm{error}(k-1)\\ xc(2)=\mathrm{error}(k)\\ xc(3)=\mathrm{error}(k)-2\mathrm{error}(k-1)+\mathrm{error}(k-2) \end{array}$$

The control algorithm is

(35) $$u_{k_{p}}(k)=k_{p}[\;\mathrm{error}\;(k)-\;\mathrm{error}\;(k-1)]$$

(36) $$u_{k_{i}}(k)=k_{i}\;\mathrm{error}\;(k)$$

(37) $$u_{k_{d}}(k)=k_{d}[\;\mathrm{error}\;(k)-2\;\mathrm{error}\;(k-1)+\;\mathrm{error}\;(k-2)]$$

(38) $$u(k)=u(k-1)+u_{k_{p}}(k)+u_{k_{i}}(k)+u_{k_{d}}(k)$$

The performance indicator function of the RBF neural network is

(39) $$E(k)=\frac{1}{2}\mathrm{error}{\left(k\right)}^{2}$$

*k_p_*, *k_i_*, and *k_d_* were adjusted using the gradient descent method:

(40) $$\Delta k_{p}=-\eta \frac{\partial E}{\partial k_{p}}=-\eta \frac{\partial E}{\partial y_{\mathrm{out}}}\frac{\partial y_{\mathrm{out}}}{\partial u}\frac{\partial u}{\partial k_{p}}=\eta \mathrm{error}(k)\frac{\partial y}{\partial u}xc$$

(41) $$\Delta k_{i}=-\eta \frac{\partial E}{\partial k_{i}}=-\eta \frac{\partial E}{\partial y_{\mathrm{out}}}\frac{\partial y_{\mathrm{out}}}{\partial u}\frac{\partial u}{\partial k_{i}}=\eta \mathrm{error}(k)\frac{\partial y}{\partial u}xc$$

(42) $$\Delta k_{d}=-\eta \frac{\partial E}{\partial k_{d}}=-\eta \frac{\partial E}{\partial y_{\mathrm{out}}}\frac{\partial y_{\mathrm{out}}}{\partial u}\frac{\partial u}{\partial k_{d}}=\eta \mathrm{error}(k)\frac{\partial y}{\partial u}xc$$

where $\frac{\partial y}{\partial u}$ is the branch output sensitivity information of the control input of the branch, or the Jacobian information of the accused object.

(43) $$\frac{\partial y}{\partial u}=\sum_{j=1}^{m}w_{j}h_{j}\frac{c_{ji}-u(k-1)}{b_{j}^{2}}$$

On the whole, the PID tuning process based on the RBF network can be presented as follows:

1. The node parameter *N* of the RBF network is first determined with the learning rate $\eta_{0}$ and inertia coefficients $\alpha_{0}$ and $\beta_{0}$ appropriately selected on the basis of the simulation analysis. Then, initial values, such as the center vector $C_{j}$, width parameter $b_{j}$, and weight coefficient $w_{j}$, are set for a series of parameters of hidden nodes.
2. The learning rate $\eta_{0}$ is clarified after initializing the number of nodes and weights of the network. Note that *k* = l at the beginning.
3. y(k) and r(k) are obtained by sampling as required, which are then included in the expression for determining e(k).
4. Three coefficients of PID are obtained after the input and output parameters of each layer are determined. On that basis, the corresponding u(k) is analyzed and calculated. Then, the obtained result is transmitted to the control object for the purpose of realizing the real-time control of the system. At the same time, the target Jacobian information is obtained through the input of the RBF identification network to obtain the output y(k+1) at the second stage.
5. The width parameters and weight coefficients of the RBF network are adjusted.
6. The weight coefficient of the BP network is adjusted.
7. Let *k* = *k* + l and return to the first step for loop iteration.

## 6. Simulation Experiment of RBF-PID Control

**Figure 11:**
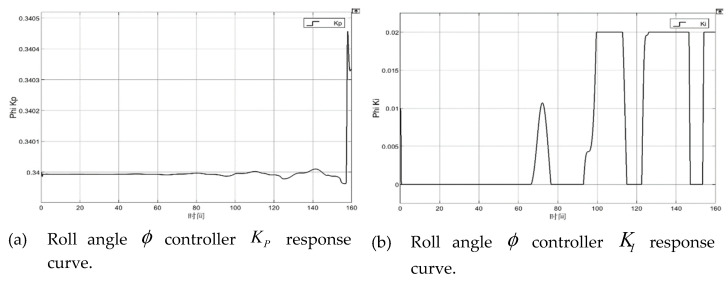

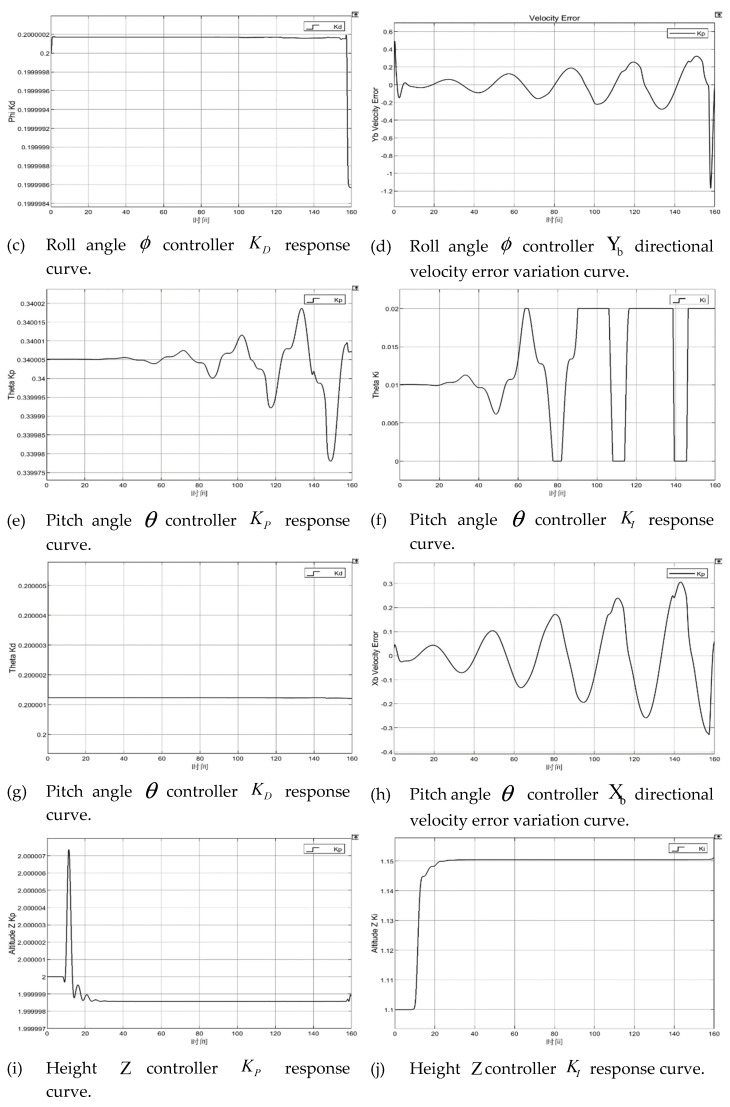

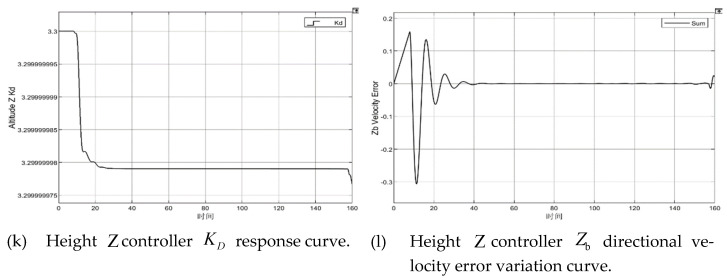
Simulation results response curves (The abscissa represents time).

The RBF-PID controller module was added to the “Theta Command Control”, the “Phi Command Control” module, and the “Altitude (Z) Control” module of the attitude controller module in the simulation platform‘s position controller module. Additionally, the “switch” module was utilized to switch with the initial PID module for the subsequent control performance comparison. The simulation time was set to 160 s, with the fixed step size t = 0.02 s as the solver sampling period; and the initial values of the $K_{p0}$, $K_{i0}$, and $K_{d0}$ parameters were $K_{p0}=0.6$, $K_{i0}=0.01$, and $K_{d0}=0.2$, respectively. The system input signal was from the path-generation module “Path Command”. Additionally, the spiral line of the preset trajectory was the desired path. The simulation results are shown in Figure 11.

By observing the roll angle φ controller, the pitch angle θ controller, and the height Z controller $K_{P}$, $K_{I}$, and $K_{D}$ response curves in the simulation of Figure 11, and with the RBF-PID controller, the $K_{P}$, $K_{I}$ and $K_{D}$ parameter values were adjusted by the RBF-PID controller according to the input error e, the error rate of change, etc. The numerical value fluctuated less. Yet, the $X_{b}$ and $Y_{b}$ direction speed error fluctuated greatly within 5 s at the end of the simulation (the endpoint hovering) (as shown in Figure 11d,h). The control performance during the flight is satisfied, with the error controlled within the range of 0.3. The error approaches 0 (as shown in Figure 11l) after the initial stage (take-off from the origin) of the speed error of the altitude Z controller in the $Z_{b}$ direction fluctuates momentarily.

**Figure 12:**
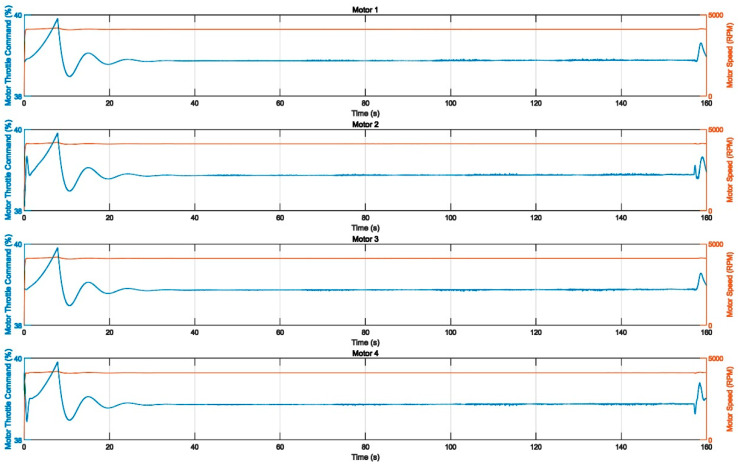
Mission UAV 1~4# motor input throttle command and output speed.

**Figure 13:**
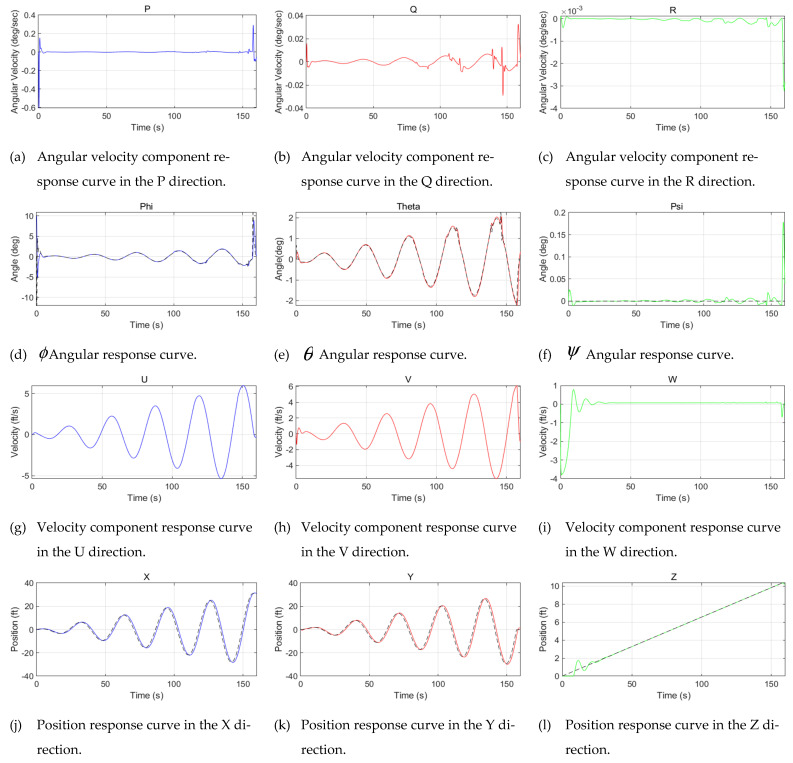
Response curves of the velocities, angles, and positions of the controller based on RBF-PID in various directions (The dashed line indicates the desired values and the colored solid line indicates the response values).

**Figure 14:**
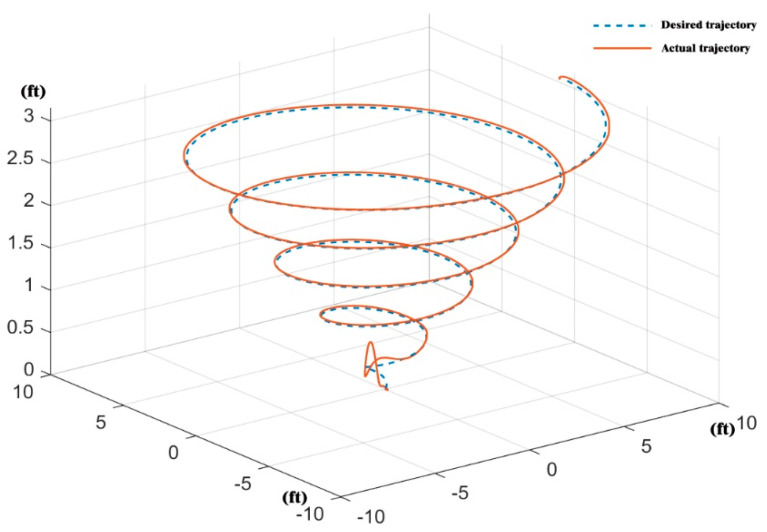
Three-dimensional trajectory tracking based on the RBF-PID controller.

The response curves of the velocities, angles, and positions of the controller based on RBF-PID in various directions were simulated, as shown in Figure 12 and Figure 13. The actual flight trajectory with high accuracy in tracking the expected trajectory can be observed from the position of the response curves in the X, Y, and Z directions. Only the initial stage of the simulation (take-off from the origin) has a slight overshoot, as shown in Figure 14, with almost no overshoot in the other stages of the process. Additionally, the mission UAV based on RBF-PID control can achieve a high-precision 3D trajectory-tracking performance.

**Figure 15:**
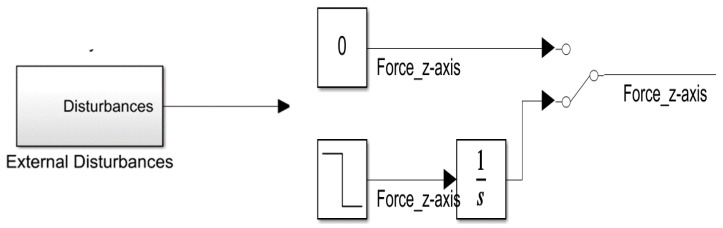
Continuous cumulative disturbance applied along the Z−axis of the geographic coordinate system of the mission UAV in the 100th second.

**Figure 16:**
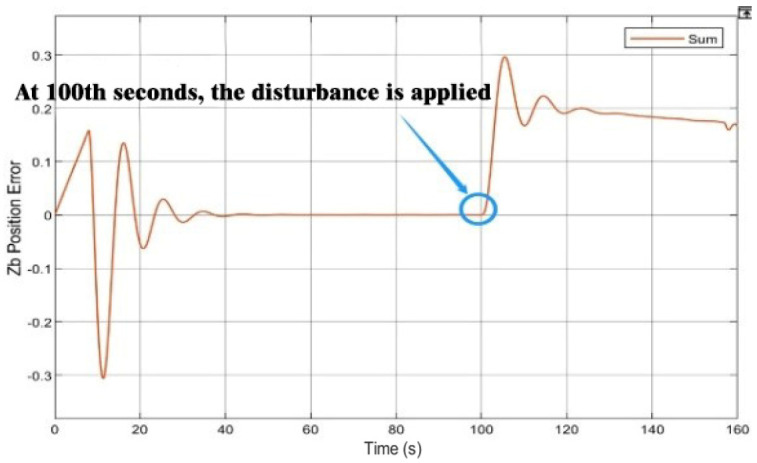
Variation curve of the velocity error along the Z−axis under the applied disturbance.

**Figure 17:**
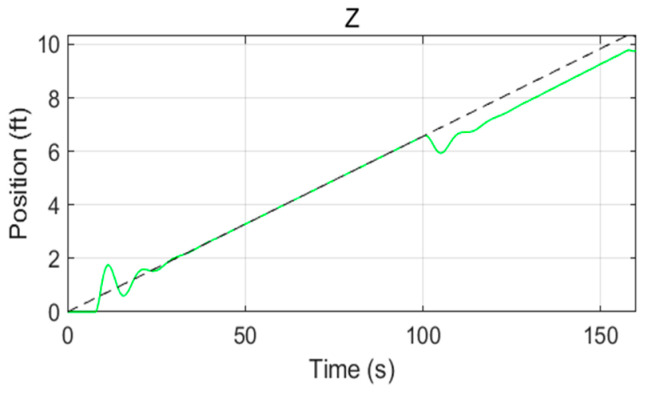
Expected trajectory and actual trajectory along the Z−axis under the applied disturbance.

The step signal module and the integral module were introduced into the simulation platform to simulate the disturbance of the blade airflow on the platform UAV because of the continuously downward increasing force of the mission UAV landing on the platform UAV, as show in Figure 15. A disturbance was applied to the design after the 100th second of the simulation cycle. Figure 16 and Figure 17 show that the variations in the velocity error increased significantly in the Z direction. The error range was maintained at 0.3 by using the RBF-PID controller. Then, the error was suppressed, gradually reduced, and controlled within 0.2 with the intervention of the RBF-PID controller. According to Figure 17, a certain error was generated between the position tracking in the Z direction and the expected trajectory at the 100th second after the continuously downward and increasing disturbance was applied, which was under control without spreading.

## 7. Design of the Vision Pilot System Based on YOLOv3

The main idea of the you-only-look-once (YOLO) system is to solve the target detection problem as a regression problem. YOLOv3 accesses the network by directly inputting the entire image and then directly regressing the frame position and the target category [17]. The YOLOv3 network divides the input image into S × S grid cells. If the geometric center of a target falls in a cell, the cell will be responsible for detecting the target. Each cell predicts the B bounding boxes and the confidence [18]. Here, B is the number of categories identified by the target in the dataset, and the confidence reflects the regression accuracy of the predicted position of the bounding box. The accuracy is the product of the probability of the target being detected and the intersection ratio of the bounding box and the real position (see Equation (44)). The confidence is defined as follows:

(44) $$\mathrm{Confidence}=P_{r}(\mathrm{Object})\;\times {\;\mathrm{IoU}}_{\mathrm{pred}\;}^{\mathrm{truth}\;}$$

where Pr(Object) is the possibility of the target existing in the box. If there is no object in the box, then Pr(Object)=0; if there is an object, then Pr(Object)=1.

**Figure 18:**
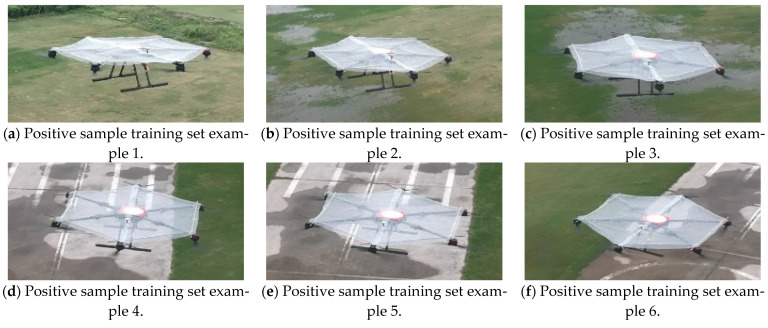

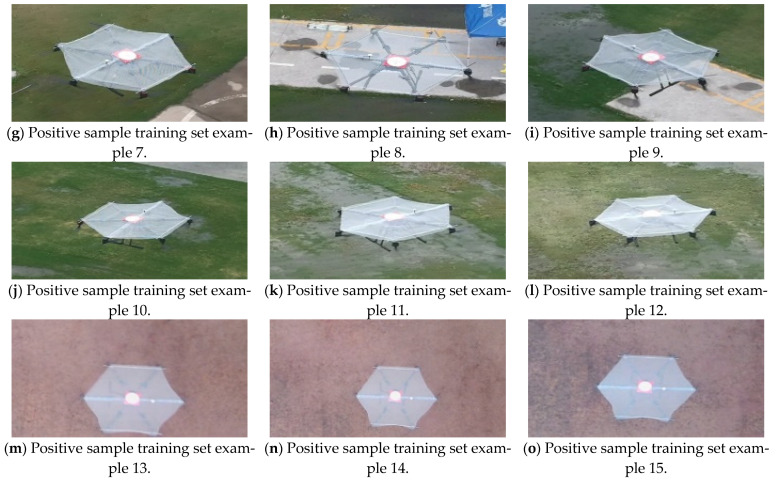
Training set of positive samples in the landing area.

The YOLOv3 network, which features a strong capability of feature expression and transfer, can learn to detect an object in the detection image upon training with large numbers of image training sets that contain a certain object [19]. We expected that YOLOv3 would detect only the target area on the platform UAV in the image detection of the video image. Therefore, the network was trained on the constructed dataset of the target area on the prepared platform where the UAV landed. Then, the trained network could detect and identify the target area in the video stream. Figure 18 shows the training set of the positive samples in the landing area.

## 8. Aerial Autonomous Docking and Landing Flight Experiment

**Figure 19:**
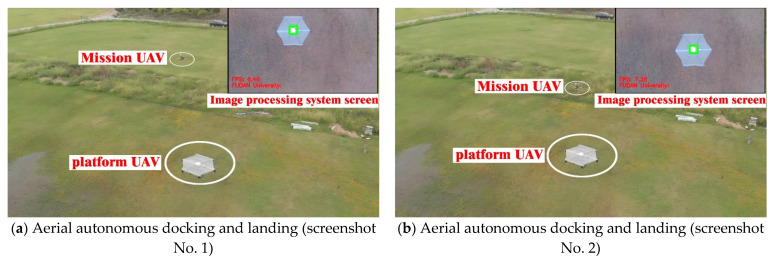

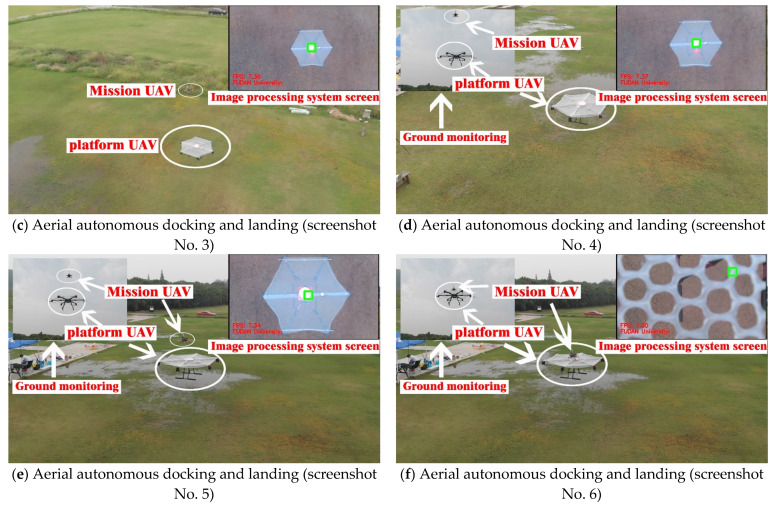
Image of aerial autonomous docking and landing test (video snapshot).

**Figure 20:**
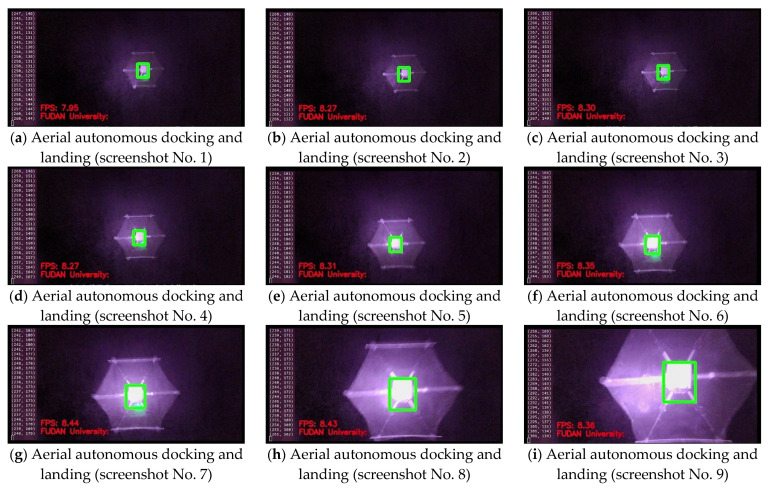

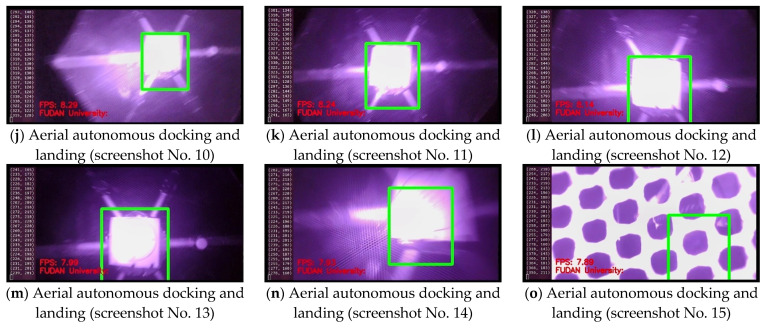
Aerial autonomous docking and landing test under infrared mode at night (video snapshot of the onboard image processing system of the mission UAV).

The designed aerial autonomous docking and landing system operated well in the field flight test. The docking and landing system recorded average system identification, positioning, and tracking during the day, which reached 8.83 FPS and 8.24 FPS, respectively, at night. The distribution of the landing points of the mission UAV on the platform UAV reached 15 cm on average in comparison with the distance accuracy from the center point of the landing surface of the platform UAV. Clearly, design expectations were satisfied in the field test. Figure 19 shows the aerial autonomous docking and landing test image in the field (see the video screenshot), and Figure 20 shows the aerial autonomous docking and landing test image under the infrared mode at night (see video snapshot of the onboard image processing system of the mission UAV).

## 9. Conclusions

This study examined the aerial autonomous docking take-off and landing technology based on multiple rotors. The precise autonomous docking and landing process control of the mission UAV is implemented on the platform UAV. First, a simulation platform is built in the MATLAB Simulink environment; the design was that of a preset, expected trajectory according to the overall design and modeling results of the mission UAV and platform UAV. Then, an autonomous docking and landing trajectory tracker based on BP-PID control was designed with the flight control influence of the rotor airflow disturbance of the platform UAV used as a key interference factor in the dual UAV’s autonomous docking and landing process. The influence of airflow disturbance on the control performance of the mission UAV was simulated with the introduction of a continuously changing disturbing quantity. The BP-PID control technology conformed with the tracking accuracy requirements of the landing trajectory under the influence of the disturbance. Moreover, a visual pilot system based on YOLOv3 was also designed to ensure that the real-time image positioning calibration rate could reach 8 FPS during the dynamic docking and landing process of the mission UAV on the platform UAV. This provides the real-time positioning requirements of the landing area where the mission UAV landed in the dynamic take-off and landing process. Finally, flight experiments were conducted. The control stability and positioning accuracy of take-off and landing proved to conform with the design expectations based on the dynamic take-off and landing tests conducted during the day and at night under the infrared mode(see Supplementary Materials). This lays a technical foundation for UAV transportation, autonomous take-off and landing in the air, and collaborative networking.

The research in this paper still has limitations, and more exploration is needed in the future.

1. In the aerial autonomous docking and landing experiment in this paper, only one mission UAV took off and landed from the platform UAV. In the future, multiple mission UAVs will take off and land together from the platform UAV, which may have new technical difficulties to be solved.

2. In the current experiment, the movement rate of the platform in flight was low, so docking and landing experiments with higher speed and complex environmental conditions are needed.

## Data Availability

This study is the contribution of author Liang Wang during his doctoral study at Fudan University and is now planned to be compiled and published. All the original experimental data and original experimental videos of this study can be obtained by contacting the corresponding author, Liang Wang. Researchers are welcome to conduct academic exchanges.

## Supplementary Materials

The following supporting information can be downloaded at: https://youtu.be/rCrgNV2GRFg, Flight experiment video.
